# Construction and evaluation of a self-replicative RNA vaccine against SARS-CoV-2 using yellow fever virus replicon

**DOI:** 10.1371/journal.pone.0274829

**Published:** 2022-10-20

**Authors:** Akina Nakamura, Tomohiro Kotaki, Yurie Nagai, Shunta Takazawa, Kenzo Tokunaga, Masanori Kameoka

**Affiliations:** 1 Department of Public Health, Kobe University Graduate School of Health Sciences, Kobe, Japan; 2 Department of Virology, Research Institute for Microbial Diseases, Osaka University, Osaka, Japan; 3 Department of Pathology, National Institute of Infectious Diseases, Tokyo, Japan; University of South Dakota, UNITED STATES

## Abstract

The coronavirus disease 2019 (COVID-19) pandemic caused by severe acute respiratory syndrome coronavirus 2 (SARS-CoV-2) infection is a global threat. To forestall the pandemic, developing safe and effective vaccines is necessary. Because of the rapid production and little effect on the host genome, mRNA vaccines are attractive, but they have a relatively low immune response after a single dose. Replicon RNA (repRNA) is a promising vaccine platform for safety and efficacy. RepRNA vaccine encodes not only antigen genes but also the genes necessary for RNA replication. Thus, repRNA is self-replicative and can play the role of an adjuvant by itself, which elicits robust immunity. This study constructed and evaluated a repRNA vaccine in which the gene encoding the spike (S) protein of SARS-CoV-2 was inserted into a replicon of yellow fever virus 17D strain. Upon electroporation of this repRNA into baby hamster kidney cells, the S protein and yellow fever virus protein were co-expressed. Additionally, the self-replication ability of repRNA vaccine was confirmed using qRT-PCR, demonstrating its potency as a vaccine. Immunization of C57BL/6 mice with 1 μg of the repRNA vaccine induced specific T-cell responses but not antibody responses. Notably, the T-cell response induced by the repRNA vaccine was significantly higher than that induced by the nonreplicative RNA vaccine in our experimental model. In the future, it is of the essence to optimize vaccine administration methods and improve S protein expression, like protection of repRNA by nanoparticles and evasion of innate immunity of the host to enhance the immune-inducing ability of the repRNA vaccine.

## Introduction

The coronavirus disease 2019 (COVID-19) pandemic caused by severe acute respiratory syndrome coronavirus 2 (SARS-CoV-2) infection has been ongoing since December 2019 until today. It has severely affected global health care and economy [1]. The primary transmission routes of SARS-CoV-2 include direct exposure by coughing, sneezing, and inhalation of droplets within a range of approximately 1.8 m [2]. It was reported that patients with mild symptoms were to recover after 10 days, whereas severe cases experienced progressive respiratory failure due to alveolar damage, likely resulting in death [3]. The incubation period ranges from 1 to 14 days, with an average of approximately 4 to 5 days [3]. Nevertheless, persons infected with SARS-CoV-2 but without symptoms can transmit it to others [4], which is known as the so-called asymptomatic or presymptomatic transmission. Thus, to curb the spread of infection, vaccine developments and vaccinations are of the essence [5, 6].

Coronaviruses are enveloped single-stranded, positive-sense RNA viruses. Four structural proteins are found in coronaviruses: spike (S), envelope, membrane, and nucleocapsid. The S protein mediates the binding of coronavirus to its receptor, angiotensin converting enzyme 2 (ACE2), expressed on the cell membrane of human alveolar epithelial cells. It is well known that anti-S neutralizing antibodies are potent in preventing coronavirus infection, and S proteins are promising as a vaccine antigen against COVID-19 [1]. Currently, all 11 vaccines that have been approved for use employ S protein as an immunogen [7, 8].

The first vaccine approved for emergency use in many countries was based on mRNA platform [9]. mRNA vaccine is an mRNA that encodes the antigen of interest. mRNA vaccines have the advantage of requiring only the genetic sequence of the target antigen, allowing for rapid and low-cost vaccine design and development. Also, they possess the advantage that the reaction is completed in the cytoplasm and has little effect on the host genome [10]. Furthermore, mRNA vaccines avoid the existing immunity problems that decrease the immunogenicity of viral vector-based vaccines. However, a single dose does not provide sufficient immunity, so they demand multiple doses over a long period to be potent [1].

Replicon RNA (repRNA) is a promising platform for vaccine generation [11]. repRNA vaccine encodes not only antigen genes but also the genes necessary for RNA replication including RNA-dependent RNA polymerase. Generally, rep RNA is a viral genome that lacks some or all of the viral structural genes. Assembly of infectious viral particles do not occur because of the absence of viral structural genes. Meanwhile, the genes necessary for the replication of viral RNA are retained, and these partial viral RNAs can replicate in the cell [12]. Because of this self-replication ability, repRNA can play the role of an adjuvant by itself and can elicit robust immune responses [1]. In several animal species, such as mice, nonhuman primates, and humans, repRNAs are potent as vaccine candidates [12–14]. Also, it has been reported that even with 1/64^th^ of the amount of RNA used, repRNA vaccines can achieve the same degree of protection as synthetic mRNA vaccines [15]. The capacity of repRNA vaccines to create robust immunity with a small dose makes them a vaccine candidate that can reduce the cost of manufacturing and rapidly end the COVID-19 pandemic [1].

Yellow fever virus (YFV) 17D strain could be an ideal replicon backbone in developing a safe and effective repRNA vaccine. YFV 17D strain has been used as a material for a live attenuated vaccine to prevent yellow fever, and its high efficacy and safety have been demonstrated since its development in 1937 [16]. YFV 17D vaccines are known to rapidly induce multifunctional immune responses, including innate, humoral, and cell-mediated immunity. Also, these characteristics are applied to vector vaccines based on the YFV 17D backbone [16, 17]. YFV 17D is used as a vector for two licensed vaccines made by replacing the gene encoding the surface antigen of YFV 17D with that of Japanese encephalitis virus (Imojev vaccine) or dengue virus (Dengvaxia vaccine) [17].

In the present study, we constructed a repRNA containing SARS-CoV-2 S protein-coding region in the YFV replicon backbone and evaluated the potency of repRNA as a COVID-19 vaccine both *in vitro* and *in vivo*.

## Materials and methods

### Cell lines and viruses

Baby hamster kidney-21 (BHK-21, hearafter BHK) cell was maintained in the Eagle’s minimum essential medium (MEM) supplemented with 10% fetal bovine serum (FBS) (Biowest) and 1% L-glutamine (Wako) at 37°C with 5% CO_2_. HEK-293T cell was maintained in the Dulbecco’s modified Eagle’s medium (DMEM) supplemented with 10% FBS at 37°C with 5% CO_2_. In addition, human osteosarcoma (HOS) cells expressing angiotensin converting enzyme 2 (ACE2) and transmembrane protease, serine 2 (TMPRSS2) (HOS/ACE2/TEMPRSS2 cells) were kept in DMEM supplemented with 10% FBS, 500 μg/mL of G418 (Nacalai Tesque), and 50 ng/mL of zeocin (Invitrogen). Also, VeroE6/TMPRSS2 cells (JCRB1819) [18] were maintained in DMEM supplemented with 10% FBS, and 1 mg/mL of G418. C6/36 cells were cultured in MEM supplemented with 10% FBS and nonessential amino acids at 28°C with 5% CO_2_. SARS-CoV-2/Hu/DP/Kng/19-020 was propagated in VeroE6/TMPRSS2 cells. The YFV 17D and dengue virus (DENV) NGC strains were propagated in C6/36 cells [19, 20]. The infectious viral titer of YFV and DENV was measured and expressed as a focus forming unit (FFU) [20].

### Construction of a plasmid encoding SARS-CoV-2 repRNA vaccine

As previously described, almost the full length of the YFV 17D genome (except 27^th^–96^th^ amino acids residue of C protein) was cloned into the pMW119 vector [21, 22]. The 27^th^–96^th^ amino acid residues of YFV-C protein were excluded to prevent the release of infectious virions. The genome was flanked with T7 promoter and hepatitis delta virus ribozyme (HDVr) sequences at the 5′ and 3′ ends, respectively. The PacI restriction enzyme site was added to the 3′ end of the HDVr. The plasmid was linearized by inverse PCR using primers, prM Vec R (5′- CTCAGATGTCACATTTAGGAGCAACCATCTG-3′) and YFV-E TM vec F (5′-ATAGGAAAGTTGTTCACTCAGACCATG-3′) designed on the prM and transmembrane region of the E proteins, respectively. SARS-CoV-2 S protein ectodomain gene was amplified using the primers YFV-C SARS-CoV-2 S F (5′-TTCGCTCCTTGTCAAACAAAATGTTTGTTTTTCTTGTTTTATTGCCACTAG-3′) and YFV-E TM SARS-CoV-2 S R (5′-TGAGTGAACAACTTTCCTATCTGCTCATACTTTCCAAGTTCTTGGAG-3′). As a template, viral RNA from the Wuhan-related prototype SARS-CoV-2 (JPN AI-I 004 strain; EPI_ISL_407084)-infected cells was used. The original sequence (neither codon optimized nor prefusion state stabilized) was used for the construction of SARS-CoV-2 vaccines in the present study [23, 24]. The linearized fragment and S protein fragment were ligated using NEBuilder HiFi DNA assembly master mix (NEB) and transformed into NEB stable competent *E*. *coli* (NEB). The plasmid (named SARS-CoV-2 S-YFV) was validated using Sanger sequencing.

### Preparation of SARS-CoV-2 repRNA vaccine

The SARS-CoV-2 S-YFV plasmid was linearized via digestion with Pac I (NEB), purified using phenol-chloroform-isoamyl alcohol (25:24:1) and chloroform sequentially, and precipitated using isopropanol. The purified DNA was washed with 70% ethanol, air-dried, dissolved in DEPC water, and used as a template DNA. Then, using the RiboMAX^™^ Large Scale RNA Production System (Promega) and m7G(5’)ppp(5’)G RNA Cap Structure Analog (NEB), the template DNA was transcribed into repRNA. The cap analog-to-GTP ratio was 1.67:1. The reaction was incubated at 37°C for 3 h. The RNA was purified using phenol-chloroform and was precipitated using isopropanol. The RNA was washed with 70% ethanol, air-dried, and dissolved in 65 μL of diethylpyrocarbonate (DEPC)-treated water. The RNA was designated as the repRNA vaccine in the present study.

### Preparation of SARS-CoV-2 non-replicative RNA vaccine

We also constructed a nonreplicative RNA (non-repRNA) encoding only the SARS-CoV-2 S protein gene essentially as described previously [25]. The S gene fragment was amplified using the forward primer including T7-promoter sequence (5′-TACTGTAATACGACTCACTATAGATGTTTGTTTTTCTTGTTTTATTGCC-3′) and the reverse primer including poly-A sequence (5′-TTTTTTTTTTTTTTTTTTTTTTTTTTCTACTGCTCATACTTTCCAAGTTCTTGG-3′). The amplified PCR fragment was transcribed into non-repRNA as described above. The cap analog-to-GTP ratio was set to 3.3:1. Then, the RNA was purified using phenol-chloroform and precipitated using isopropanol. Finally, the extracted RNA was washed with 70% ethanol, air-dried, and dissolved in 20 μL of DEPC-treated water. The RNA was designated as the non-repRNA vaccine in the present study.

### RNA electroporation

The repRNA and non-repRNA vaccines were electroporated into BHK cells using NEPA21 electroporator (Nepagene). The BHK cells were trypsinized and washed twice with Opti-MEM (Thermo Fisher Scientific). The washed cells (1 × 10^6^) were mixed with 10 μg of repRNA vaccine or 3 μg of non-repRNA vaccine (equivalent genome copies to 10 μg of repRNA vaccine) in 100 μL of Opti-MEM. Electric pulses were given using NEPA21 electroporator. The parameters for BHK cells were as follows: voltage = 145 V; pulse length = 5 milliseconds (ms); pulse interval = 50 ms; number of pulses = 1; decay rate = 10%; polarity + as poring pulse and voltage = 20 V; pulse length = 50 ms; pulse interval = 50 ms; number of pulses = 5; decay rate = 40%; and polarity +/− as transfer pulse. After electroporation, the cells were seeded as 1.0 × 10^5^ cells/well in a 24-well plate.

### Western blotting

The expression of S protein in BHK cells was confirmed using western blotting. At 48 h post transfection, cells were harvested, solubilized in SDS-PAGE sample buffer, developed into 10% polyacrylamide gel using SDS-PAGE, and transferred to a membrane. Anti-SARS-CoV/SARS-CoV-2 Spike, Mouse-Mono (1A9, GeneTex), or anti-β-actin mouse monoclonal antibody (Sigma-Aldrich) was used as a primary antibody, and anti-mouse HRP was used as a secondary antibody. Antibody 1A9 targets S2 region of the S protein. Then, chemiluminescence was induced using ImmunoStar LD (FUJIFILM). The band intensity was quantified using the ImageJ software [26].

### Immunofluorescence assay

The co-expression of S protein and YFV protein was confirmed using immunofluorescence assay. At 48 h post transfection, the cells were fixed with 4% paraformaldehyde and permeabilized with 0.5% Triton-X. After blocking with 1% normal goat serum, the cells were incubated with the primary monoclonal antibodies (mAbs) [Anti-SARS-CoV/SARS-CoV-2 Spike, Mouse-Mono (GeneTex) and Anti-YFV NS1 Protein, Rabbit-Poly (GeneTex)]. Then, the secondary antibodies [goat anti-mouse IgG conjugated with Alexa Flour 488 (abcam) and goat anti-rabbit IgG conjugated with Alexa Fluor 568 (abcam)] were added. After incubation, the cells were mounted in a mounting medium containing 4′,6-diamidino-2-phenylindole (DAPI: Vector Laboratories). A fluorescence microscope was used for fluorescence imaging (ZOE Fluorescent Cell Imager, BIO-RAD).

### Real-time RT-PCR

Real-time RT-PCR was performed to verify the self-replication ability of the repRNA vaccine in BHK cells. BHK cells electroporated with the repRNA or non-repRNA vaccine were collected at 2, 6, 24, 48, 72, and 96 h post transfection. Intracellular RNA was extracted using ISOSPIN Cell & Tissue RNA (NIPPON GENE). Real-time RT-PCR assays were performed using forward primer (5′-CCTACTAAATTAAATGATCTCTGCTTTACT-3′), reverse primer (5′-CAAGCTATAACGCAGCCTGTA-3′), probe (5′-FAM-CGCTCCAGGGCAAACTGGAAAG-BHQ-3′), and THUNDERBIRD^®^ Probe One-step qRT-PCR Kit (TOYOBO) in a CFX Connect Real-Time PCR Detection System (BIO-RAD). Using non-repRNA vaccine, a series of 8 log_10_ dilutions equivalent to 1 × 10^−7^ to 1 ng per reaction mixture were prepared to generate calibration curves and were run in parallel with the test samples.

### Mouse immunizations

The institutional Animal Experiment Committee (Ethics Committee Approval Number: P180504) approved all animal experiments at Kobe University Graduate School of Health Sciences. Six-week-old female C57BL/6 mice (n = 5–6/group) (SLC) were immunized with 1 μg of repRNA vaccine, 1 μg of non-repRNA vaccine, or phosphate-buffered saline (PBS) via the intratibial route on days 0, 28, and 56. All RNA vaccines were diluted in 100 μL of 1× PBS. Before initial immunization, 100 units of hyaluronidase were injected into the mice’s thighs and left for 30 min. Electric pulses were provided to mice’s thighs using NEPA21 electroporator to enhance the delivery of RNA vaccines. The parameters for mice immunization were as follows: voltage = 80 V; pulse length = 30 ms; pulse interval = 50 ms; number of pulses = 3; decay rate = 10%; polarity + as poring pulse and voltage = 20 V; pulse length = 50 ms; pulse interval = 50 ms; number of pulses = 3; decay rate = 40%; and polarity +/− as transfer pulse. Mice immunization and euthanasia by cervical dislocation were performed under anesthesia using isoflurane inhalation. A trained laboratory personnel performed all the animal experiments.

### SARS-CoV-2 virus culture, purification, and inactivation

All experiments containing infectious SARS-CoV-2 were performed in the biosafety level (BSL)-3 facility of Kobe University Graduate School of Medicine. VeroE6/TMPRSS2 cells were grown in T75 flasks to a confluence of approximately 80%–90% and were infected with SARS-CoV-2 (SARS-CoV-2/Hu/DP/Kng/19-020). After 1 h at 37°C, 15 mL of DMEM with 10% FBS was added, and cells were incubated for 24 hour at 37°C with 5% CO_2_. The viral supernatant was chemically inactivated with 0.03% β-propiolactone (Wako) at 4°C for 24 h. β-Propiolactone was hydrolyzed at 37°C for 2 h. Inactivated virus was purified using ultracentrifugation on a 20% sucrose cushion at 30,000 rpm for 2 h at 4°C. Finally, the pellet was resuspended in PBS. Inactivation of the virus was confirmed by measuring median tissue culture infectious doses (TCID_50_) using VeroE6/TMPRSS2 cells.

### ELISA

To detect IgG-antibodies to SARS-CoV-2 S, an enzyme-linked immune-sorbent assay (ELISA) using inactivated virus was performed. Shortly, the 96-well flat-bottomed MaxiSoap plates (Thermo Fisher Scientific) were coated with 36.4 ng/mL of inactivated SARS-CoV-2 viral particles in 100 μL per well in carbonate coating buffer (15 mM Na_2_CO_3_, 7 mM NaHCO_3_, pH 9.6) overnight at 4°C. All washes were performed three times using PBS containing 0.05% Tween, and all incubations were performed for 1 h at 37°C. The coated plates were washed and blocked by incubating with 1% bovine serum albumin in PBS. Then, the plates were serially incubated with 100 μL per well of diluted mouse antiserum (1:200) and 100 μL per well of alkaline phosphatase (AP)-conjugated Goat Anti-Mouse IgG (H+L) (1:2000) (Jackson Immuneresearch). Next, disodium p-Nitrophenylphosphate Hexa-hydrate (Wako) was added and incubated for 40 min for color development. Absorbance was detected at 415 nm in a microplate reader (iMark Microplate Absorbance Reader, BIO-RAD). All measurements were performed in duplicate.

In addition, an ELISA was performed to detect antibodies against YFV nonstructural protein 1 (NS1), which is included in the replicon backbone. The maxisorp plate was coated with anti-YFV NS1 protein (1:2000) overnight at 4°C. Then, culture fluid from the YFV-infected cells (1 × 10^5^ FFU/well), immunized mouse sera (1:200), AP-conjugated anti-mouse IgG were serially incubated, followed by color development.

### SARS-CoV-2 pseudovirus preparation

S protein-pseudotyped, luciferase reporter lentivirus was prepared as a tool to evaluate humoral immunity in mice. As previously described, to prepare the pseudovirus, 3.0 × 10^5^ 293T cells were co-transfected with 400 ng of spike protein expression plasmids (pCA SARS-CoV-2 S D614G), 800 ng of psPAX2-IN/HiBiT, and 800 ng of pWPI-Luc2, using FuGENE^®^ HD Transfection Reagent (Promega) [27]. After 48 h post transfection, the viral titers in supernatants were measured using the HiBiT assay as follows. Lentiviral stocks containing known levels of HIV-1 Gag p24 antigen were serially diluted as standards. According to the manufacturer’s protocols, standards or pseudoviruses (25 μL) and Nano-Glo HiBiT Lytic Buffer (25 μL) containing LgBiT Protein (1:100) and HiBiT Lytic Substrate (1:50) (Nano-Glo HiBiT Lytic Detection System; Promega) were mixed and incubated for 10 min at room temperature. The HiBiT-based luciferase activity in the viral supernatant was measured using a CentroPRO LB962 luminometer (Berthold) and was converted to p24 antigen levels.

### SARS-CoV-2 pseudovirus neutralization assay

HOS/ACE2/TMPRSS2 cells were seeded at 2.0 × 10^4^ cell/well in 96-well plates for 16–24 h. Pseudovirus of 2 ng was incubated with 100 μL of serially diluted mouse antiserum at 37°C for 1 h. The pseudovirus/antiserum mixture was replaced with the cell culture medium. After 48 h of incubation, the luciferase activity was measured using a Nano-Glo HiBiT Lytic Detection System (Promega) and a CentroPRO LB962 luminometer (Berthold).

### Evaluation of repRNA vaccine containing DENV envelope gene

To validate the adequacy of the YFV replicon backbone system and *in vivo* RNA electroporation for immunization, the YFV replicon containing envelope (E) gene of DENV was constructed and evaluated in essentially the same way as the SARS-CoV-2 repRNA vaccine. Briefly, the plasmid containing YFV 17D genome (except 27^th^–96^th^ amino acids residue of C protein) was linearized via inverse PCR using a forward primer (5′-GATCAAGGATGCGCCATCAACTTTGGC-3′) and a reverse primer (5′-TGAGTAGGCCGGACCAACAGCC-3′). DENV E protein gene was amplified using the following forward and reverse primers, respectively: 5′-GGTCCGGCCTACTCAATGCGTTGCATAGGAATATC-3′ and 5′-GGCGCATCCTTGATCGGCCTGCACCATAACTCCCAAATAC-3′. The two fragments were ligated and cloned into *E*. *coli*, followed by *in vitro* RNA transcription as described above. This is designated as the dengue repRNA vaccine. E gene expression *in vitro* was confirmed using an immunofluorescence assay using anti-E antibody (D1-4G2, ATCC). Self-replicability was examined via real-time RT-PCR using the following forward and reverse primers, respectively: 5′-AGCATGCAGTCGGAAATGAC-3′ and (5′-CAGTGCCATAGCCTGTCAAC-3′). The probe used was 5′-’FAM-CGCTCCAGGGCAAACTGGAAAG-BHQ-3′. BALB/c mice were immunized with the dengue repRNA as described above. Serum samples were harvested after second immunization. Antibody production in mice was examined using ELISA. The maxisorp plate coated with 1 × 10^5^ FFU/well of DENV was serially incubated with immunized sera (1:200) and AP-conjugated anti-mouse IgG, followed by color development.

### Mouse IFN-γ ELISpot assay

At 20 days after the third vaccination, spleen lymphocytes were isolated from mice. Using these splenocytes, enzyme-linked immunosorbent spot (ELISpot) was performed according to the manufacturer’s protocols using the Murine IFN-γ single-color Enzymatic ELISPOT assay (IMMUNOSPOT). First, the splenocytes (3 × 10^5^ cells/well) seeded onto assay plates were stimulated with inactivated SARS-CoV-2 virus particles as described above (0.364 μg/mL). IFN-γ-secreting cells were detected after 24 h of incubation. Then, using an Immunospot Analyzer (CTL), spot forming cells were counted.

### Statistical analysis

Statistical analyses were performed using the standard function of GraphPad Prism 8 software (GraphPad Software) with Student’s t-test, one-way analysis of variance (ANOVA), or two-way ANOVA.

## Results

### Construction and characterization of repRNA

**Fig 1:**
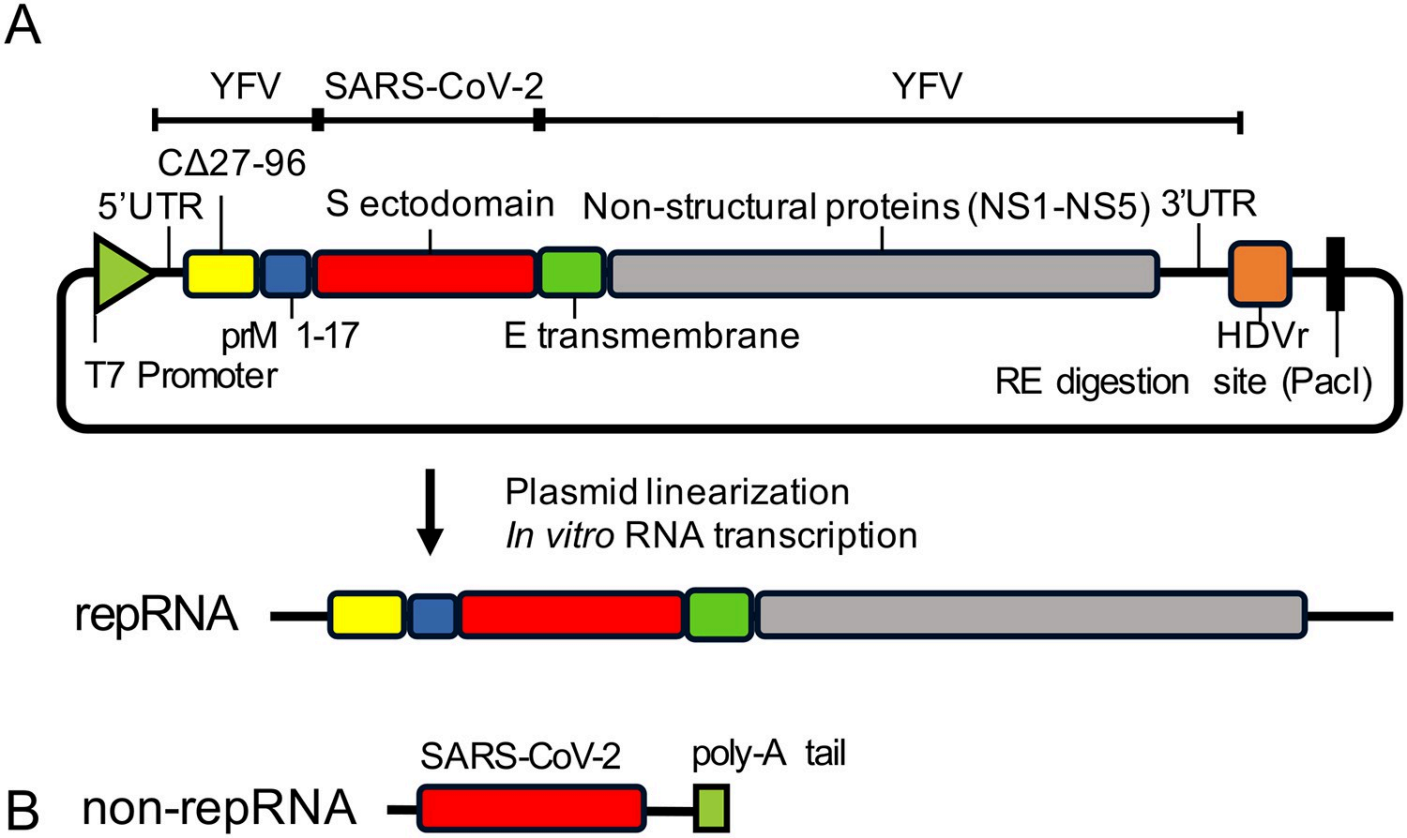
Construction of repRNA and non-repRNA vaccines. (A) Structure of SARS-CoV-2 repRNA vaccine. SARS-CoV-2 S protein ectodomain gene was inserted into the YFV replicon encoding seven nonstructural proteins (NS1–5) and three structural proteins [C (lacking 27^th^–96^th^ amino acid), prM (1^st^–17^th^ amino acid), and E (transmembrane region)] genes of the YFV 17D strain. The T7 promoter and HDVr sequences were added to the genome’s 5′ and 3′ ends, respectively. PacI site was added to the 3′ end of the HDVr. (B) Structure of non-repRNA vaccine. A non-repRNA vaccine was transcribed from a PCR product encoding T7 promoter sequence, SARS-CoV-2 S gene, and poly-A sequence.

We generated a DNA plasmid encoding the repRNA vaccine that contains the ectodomain of S protein in the YFV replicon backbone (Fig 1A). The repRNA vaccine was transcribed from the DNA plasmid linearized with the PacI restriction enzyme (Fig 1A). In addition, a non-repRNA vaccine for control was transcribed from a PCR product encoding T7 promoter sequence, SARS-CoV-2 S gene, and poly-A sequence (Fig 1B).

**Fig 2:**
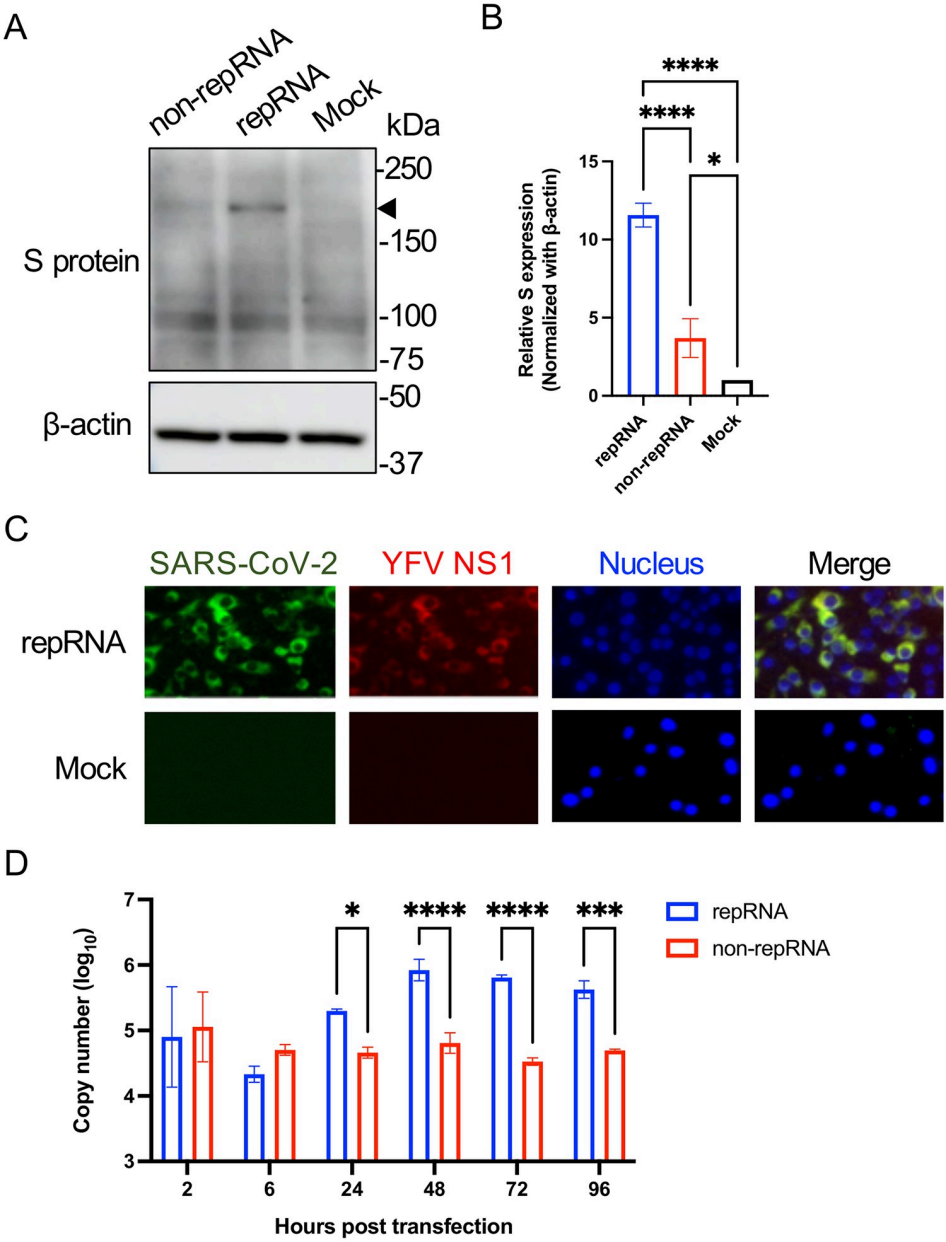
Characterization of repRNA and non-repRNA vaccines *in vitro*. (A) Confirmation of SARS-CoV-2 S protein expression via western blotting. BHK cells were electroporated with either the repRNA or non-repRNA vaccine and subjected to western blotting at 48 h post transfection. The triangle indicates the full-length S protein (approximately 180 kDa). Original unedited blot images are shown in the S1 Raw image. (B) Quantification of the S protein. The band intensity was quantified using the ImageJ software. The S protein expression level was normalized by β-actin. p-values were determined using one-way ANOVA, and p < 0.05 was considered significant (*p < 0.05, ****p < 0.0001). (C) The detection of S protein and YFV NS1 protein using immunofluorescence assay. BHK cells were electroporated with 10 μg of repRNA vaccine. At 48 h post transfection, the cells were fixed with 4% paraformaldehyde, followed by permeabilization with 0.5% Triton-X. The expression of S protein was detected using anti-S mAb and goat-anti-mouse IgG conjugated with Alexa Fluor 488; YFV NS1 protein expression was detected using anti-NS1 pAb and goat-anti-rabbit IgG conjugated with Alexa Fluor 568. Cell nuclei were stained with DAPI. Untransfected BHK cells were used as a negative control. (D) Kinetics of the RNA levels. BHK cells were electroporated with repRNA or non-repRNA vaccines. RNA copy numbers were then measured using qRT-PCR. p-values were determined using two-way ANOVA, with p < 0.05 considered to be significant (*p < 0.05, ***p < 0.001, ****p < 0.0001). Statistically significant values are represented in the graphic.

To determine the protein expression *in vitro*, the RNAs were directly electroporated into BHK cells. Intracellular expression of the S protein by both repRNA and non-repRNA was confirmed via western blotting (Fig 2A). The S protein expression by repRNA was significantly higher than that by non-repRNA, which showed barely detectable S protein (Fig 2B). Only the full-length S protein was detected via western blotting (Fig 2A). The S2 band, which is generally fainter than the full-length S band, was undetectable [28]. In addition, the S protein was not detected in the culture supernatant of the transfected cells via western blotting (data not shown). To further characterize the repRNA vaccine, the co-expression of S protein and YFV-NS1 by rwas confirmed via an immunofluorescence assay (Fig 2C). Also, the kinetics of the RNA levels in the transfected cells was examined using real-time RT-PCR to investigate the self-replication ability of the repRNA vaccine. At 24–96 h post transfection, a significant increase in RNA level was observed in cells transfected with repRNA vaccine compared with cells transfected with non-repRNA vaccine (Fig 2D). These data demonstrated that the repRNA vaccine was successfully constructed and self-replicative, demonstrating its vaccine potency.

### Immunogenicity of repRNA in C57/BL6 mice

**Fig 3:**
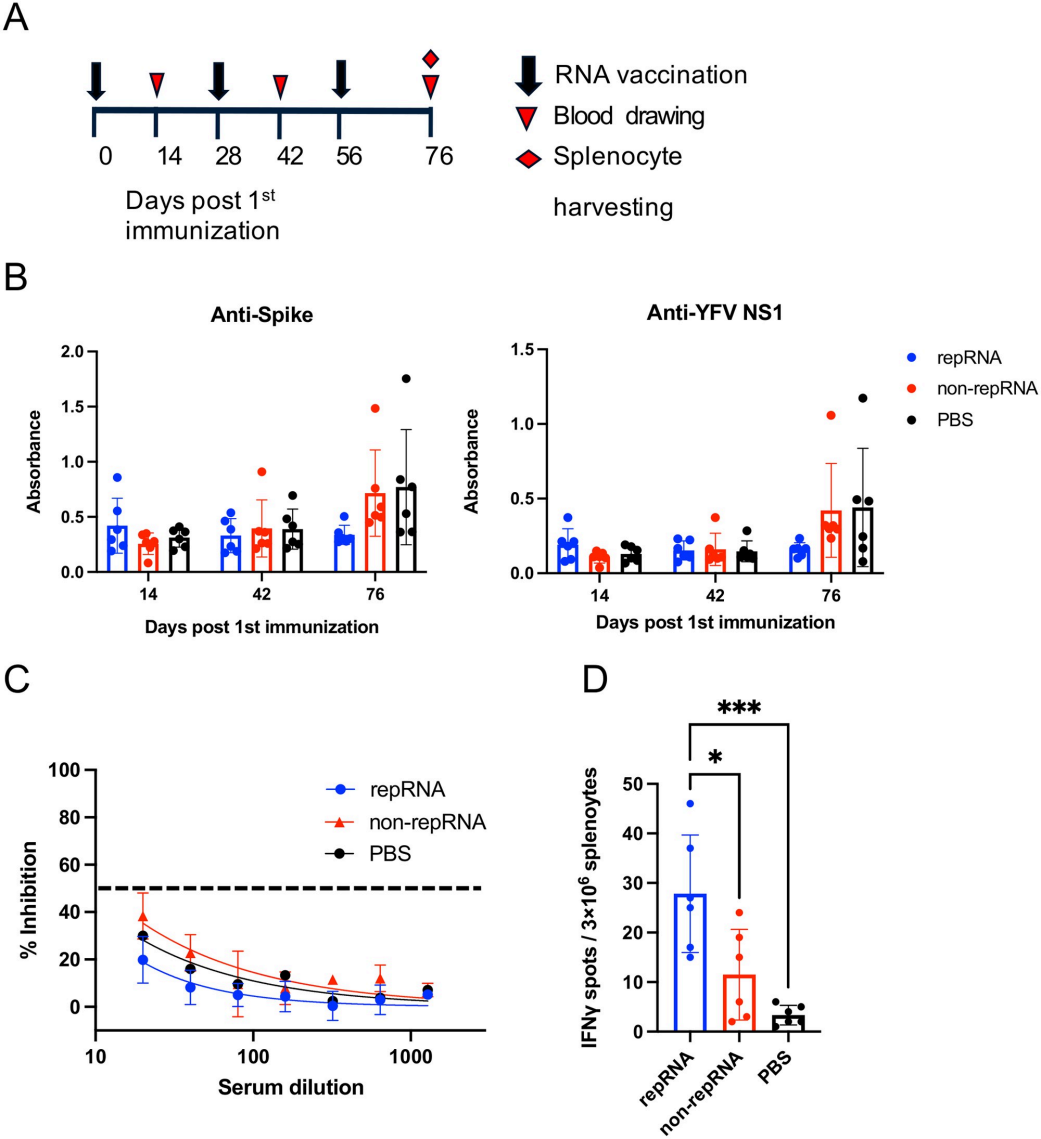
Evaluation of the repRNA and non-repRNA vaccines *in vivo*. (A) Immunization schedule for mice. Six-week-old C57BL/6 mice were immunized with 1-μg repRNA vaccine, 1-μg non-repRNA vaccine, or PBS (n = 6–7) via intratibial route with electroporation. Serum and/or splenocytes were harvested at 14, 42, and 76 days after the first immunization. (B) Measurement of anti-S IgG and anti-YFV NS1 antibody level. (C) Neutralizing activity in mouse sera. Neutralizing activity was measured using SARS-CoV-2 pseudovirus neutralization assay. Luminescence was measured at 48 h after pseudovirus infection. The dotted line indicates the 50% neutralization. (D) ELISpot assay using splenocytes. On day 76, splenocytes were harvested, and IFN-γ response was measured using ELISpot assay. p-values were determined using one-way ANOVA, with p < 0.05 considered to be significant (*p < 0.05, ***p < 0.001). Statistically significant values are represented in the graphic.

C57BL/6 mice were immunized with 1 μg of repRNA vaccine, 1 μg of non-repRNA vaccine, or PBS three times at 4-week intervals, and the serum and splenocytes were used to evaluate the immunogenicity of the vaccines (Fig 3A). IgG antibody titer against the S protein and YFV-NS1 was measured using ELISA. However, there was no significant difference between the groups (Fig 3B). In addition, neutralizing activity was analyzed using a pseudovirus neutralization assay (Fig 3C). Once again, the neutralizing activity was not detected (i.e., less than 50% inhibition even at the lowest serum dilution). Taken together, humoral immunity was not robustly induced by both the vaccines.

To validate the adequacy of the YFV replicon system and *in vivo* RNA electroporation as an immunization strategy, the YFV replicon containing E gene of DENV was constructed and evaluated in essentially the same way as the SARS-CoV-2 repRNA vaccine (S1A Fig in S1 File). The dengue repRNA vaccine expressed E protein in BHK cells and was self-replicative (S1B, S1C Fig in S1 File). The dengue repRNA vaccine induced detectable antibodies against E protein in mice (S1D Fig in S1 File). Altogether, the YFV replicon system and immunization method were robust enough to induce humoral immunity. In other words, the low humoral immunity induced by the SARS-CoV-2 repRNA vaccine was attributed to the RNA construct itself.

To further investigate the induction of cellular immunity, an ELISpot assay was performed. Splenocytes from mice in the cohort of SARS-CoV-2 vaccination were harvested 20 days after the third immunization, and subjected to ELISpot assay. Mice injected with 1 μg of repRNA vaccine, 1 μg of non-repRNA vaccine, or PBS showed responses in splenic T-cells, with mean IFN-γ spots per 10^6^ splenocytes of 10.3, 8.3, and 1.8, respectively (Fig 3D). The number of IFN-γ spots in repRNA vaccine group was significantly higher than that in non-repRNA vaccine and PBS groups. These results suggested that the repRNA vaccine induced T-cell responses in mice, although it was limited, and the response was more robust compared with that of the non-repRNA vaccine in our experimental model.

## Discussion

The SARS-CoV-2 pandemic is a global threat, demanding the development of more effective and safer vaccines to curb the spread of infection. Using the YFV 17D strain backbone, we report on constructing a repRNA vaccine against COVID-19. repRNA vaccines are promising vaccine candidates that have already been registered to demonstrate exemplary safety and immunogenicity [29].

In the present study, both repRNA and non-repRNA vaccines expressed the S protein of SARS-CoV-2 in electroporated BHK cells. The repRNA vaccine showed significantly higher expression of the S protein than the non-repRNA vaccine (Fig 2B). In addition, an increase in the level of repRNA was confirmed in the transfected cells (Fig 2D). These results indicate the potency of repRNA in the present study. However, only the full-length S protein was detected (Fig 2A). In addition, the S protein was not detected in the culture supernatant of the transfected cells via western blotting (data not shown). These data indicate that the level of S protein expression was low, even in the repRNA-transfected cells.

Concerning humoral immunity in mice, there was no significant difference in the IgG antibody induction between the RNA vaccine-treated groups and the negative control PBS-treated group. Erasmus *et al*. reported that immunization of mice with 1 μg of the repRNA vaccine against SARS-CoV-2 induced an IgG antibody titer of 937 μL/ml and a 50% inhibitory concentration of 1:226, indicating a high level of neutralization activity [1]. Considering the fact that the dengue repRNA vaccine induced detectable humoral immunity against DENV in our experimental system (S1 Fig in S1 File), the low immunogenicity of the SARS-CoV-2 repRNA vaccine is attributed to the RNA construct itself.

There are several possible reasons for the low induced immunity in the present study. Considering that the S protein level was low even in the repRNA-transfected cells, the difference in codon usage is one possible reason. We used the original sequence (i.e., neither codon optimized nor prefusion state stabilized) of SARS-CoV-2 S gene in the repRNA and non-repRNA vaccines. The codon usage pattern of SARS-CoV-2 is almost antagonistic to human codon usage patterns [30]. The antagonism between viral and host codon usage may result in slow viral mRNA translation, resulting in low S protein expression [31]. Codon optimization of the vaccine sequence is a promising approach to improve immunogenicity [1, 31]. In addition, stabilization of the translated S protein to prefusion state by prorine substitutions to K986 and/or V987 or furin cleavage sequence knockout could be applied to improve immunogenicity [23, 24]. Another possible reason is the instability of the repRNA itself. Naked repRNAs are labile molecules; a single cleavage would severely impair translation and abolish self-replication [32]. Before administration, the RNA vaccine was protected by lipid nanoparticles in the study [1]. This difference may have led to the difference in results between the present study and those of the previous study [1]. Also, it is possible that the natural immunity of the mice worked against the repRNA vaccine. It has been reported that the expression and induction of humoral responses by self-replicative RNA vaccines are significantly lower in wild type mice than in IFNAR1 −/− mice [33]. Additionally, it has been suggested that inducible IFNs reduce the expression of self-replicative RNA vaccines by inhibiting translation and negatively affecting the elicited humoral immune response [34, 35]. It was reported that the vaccine encoding Npro, which selectively degrades interferon regulatory factor-3 (IRF-3), induced more immunity by avoiding the influence of IFNs [36]. Also, nucleoside modification, such as pseudouridylation, could be used to enhance mRNA translation by evading foreign RNA sensing and following IFN activation in host cells [37, 38]. In improving the immunogenicity of repRNA vaccines, strategies like sequence optimization, use of nanoparticles for vaccine protection, or reducing the type I IFN activation of the repRNA itself should be considered.

ELISpot results showed that the number of formed spots in spleen cells of mice vaccinated with 1 μg of repRNA vaccine was significantly higher than that of PBS and 1 μg of non-repRNA vaccine groups. This suggested that the repRNA vaccine produced in this study induced T-cell responses in mice, although it was limited. Furthermore, the repRNA vaccine induced higher cellular immunity than the non-repRNA vaccine used in the present study. In immunity against SARS-CoV, it has been reported that T-cell immunity contributes more to long-term protection than B-cell immunity [39] and that SARS-CoV-2-specific T-cell responses are associated with better recovery from COVID-19 [40]. This suggested the higher potency of repRNA vaccine than non-repRNA vaccine at least in our experimental model. In the future, tests are needed to confirm the existence of a long-term immune response to the repRNA vaccine.

The difference between humoral immunity and cellular immunity stimulations could be attributed to the expression pattern of S protein. The intracellular antigen is presented by MHC class I and stimulates cellular immunity, whereas the extracellular antigen is presented by MHC class II and stimulates humoral immunity [41, 42]. This study expressed S protein as a fused protein with prM (1^st^–17^th^ amino acids) and E (transmembrane region) of YFV. Although we have confirmed the intracellular S protein expression (Fig 2A–2C), extracellular S protein was not detected via western blotting (data not shown). This could be due to the low expression of the S protein and/or its inefficient secretion, leading to the low stimulation of MHC class II and thereby low humoral immunity induction. Further improvement of not only the S sequence but also the YFV replicon vector itself, such as addition of a protein secretion signal peptide and/or self-cleaving peptide, should be considered.

In the present study, BHK cells were used for the *in vitro* characterization of repRNA and non-repRNA vaccines because the cells have been commonly used to construct coronavirus replicon and coronavirus protein expression [43, 44]. Nevertheless, it should be noted that S protein expression and self-replication ability *in vitro* are not directly reflected in the trend of repRNA expression *in vivo* because BHK cell is defective in IFN production [45]. It should also be noted that the non-repRNA vaccine in this study had no untranslated region and short poly-A tail, which may attenuate protein expression level. Although the effect of RNA construct design on protein expression level needs to be considered, the results validate the superiority of repRNA at least in our experimental model.

Summarily, this study confirmed that the repRNAs produced could express S protein and self-replicative ability *in vitro*. Nonetheless, the vaccine did not robustly induce humoral immunity in mice, probably due to low S protein expression. Notwithstanding, there was a tendency to generate a specific T-cell response to S protein. The degree of T-cell activation induced by the repRNA vaccine was significantly higher than that induced by the non-repRNA vaccine at least in our experimental model, indicating the usefulness of repRNA as a vaccine. However, further optimization of vaccine administration method and improvement of S protein expression, like sequence optimization, protection of repRNA by nanoparticles, and evasion of innate immunity of the host, are required to improve the immune-inducing ability of the repRNA vaccine.

## Supporting information

S1 File (PDF)

S1 Raw image (PDF)
