# Supplementary material for: Construction and evaluation of a self-replicative RNA vaccine against SARS-CoV-2 using yellow fever virus replicon
Source: PLoS One. 2022 Oct 20;17(10):e0274829. doi: 10.1371/journal.pone.0274829 (PMC9584447; doi:10.1371/journal.pone.0274829)
Supplement: S1 File — (PDF) [file pone.0274829.s001.pdf]

Supporting information

Construction and evaluation of a self-replicative RNA vaccine against SARS-CoV-2 using yellow fever virus replicon

Akina Nakamura[1¶], Tomohiro Kotaki[1,2¶*], Shunta Takazawa[1], Yurie Nagai[1], Kenzo Tokunaga[3], Masanori Kameoka[1*]

[1] Department of Public Health, Kobe University Graduate School of Health Sciences, Kobe, Japan

[2] Department of Virology, Research Institute for Microbial Diseases, Osaka University, Osaka, Japan

[3] Department of Pathology, National Institute of Infectious Diseases, Tokyo, Japan.

[*] Corresponding authors:

Tomohiro Kotaki (tkotaki@biken.osaka-u.ac.jp)

Masanori Kameoka (mkameoka@port.kobe-u.ac.jp)

[¶]These authors contributed equally to this work.

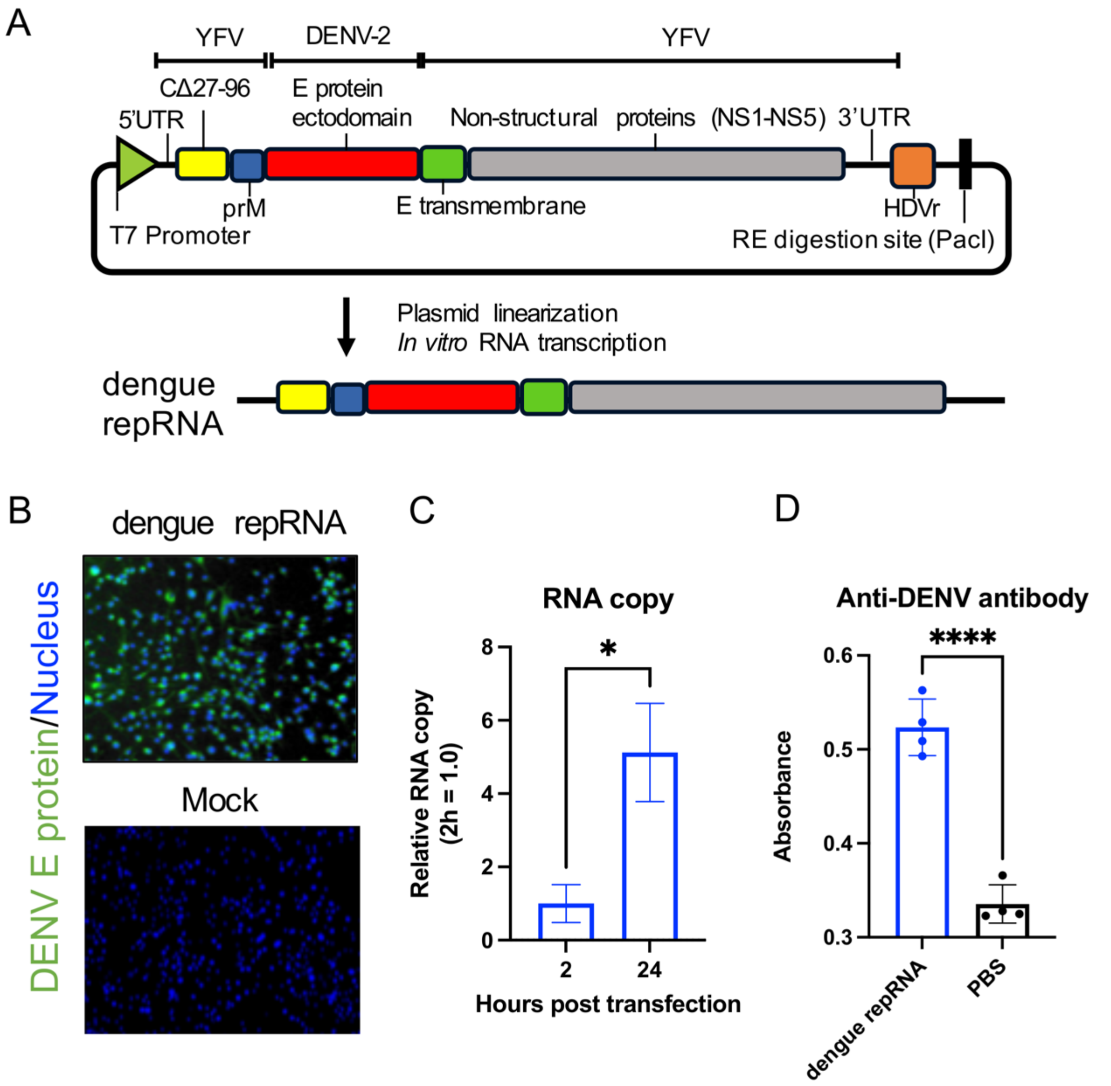

Supplementary figure S1. Characterization of dengue repRNA vaccine *in vitro* and *in vivo*

(A) Structure of dengue repRNA vaccine. (B) Confirmation of DENV E protein expression by immunofluorescence assay. BHK cells were electroporated with 10 µg of dengue repRNA vaccine. At 24 h post-transfection, the cells were fixed with 4% paraformaldehyde, followed by permeabilization with 0.5% Triton-X. The expression of E protein was detected using anti-E mAb and goat-anti-mouse IgG conjugated with Alexa Fluor 488. Cell nuclei were stained with DAPI. Untransfected BHK cells were used as a negative control. (C) Kinetics of the dengue repRNA level. BHK cells were electroporated with dengue repRNA vaccine. Relative RNA copy numbers (normalized to 2 h post-transfection sample) were then measured using qRT-PCR. p-value was determined using t test, with $p < 0.05$ considered to be significant (*$p < 0.05$). (D)

Measurement of anti-E IgG antibody level. Mice antiserum was used for ELISA. p-value was determined using t test, with $p < 0.05$ considered to be significant (****$p < 0.0001$).
