# Supplementary material for: Construction and evaluation of a self-replicative RNA vaccine against SARS-CoV-2 using yellow fever virus replicon
Source: PLoS One. 2022 Oct 20;17(10):e0274829. doi: 10.1371/journal.pone.0274829 (PMC9584447; doi:10.1371/journal.pone.0274829)

Original blot images

Framed area indicated the cropped image as seen in the Figure 2A. No figures were cropped and joined together from different images. Contrast adjustment was applied equally across the entire image.

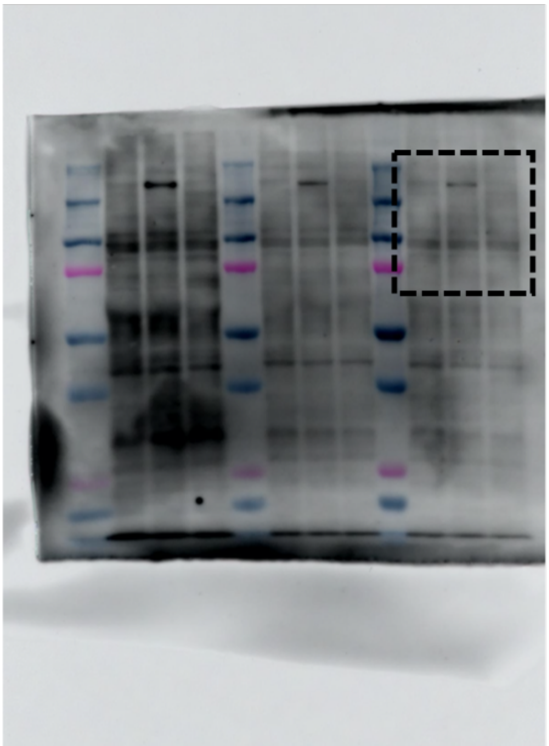

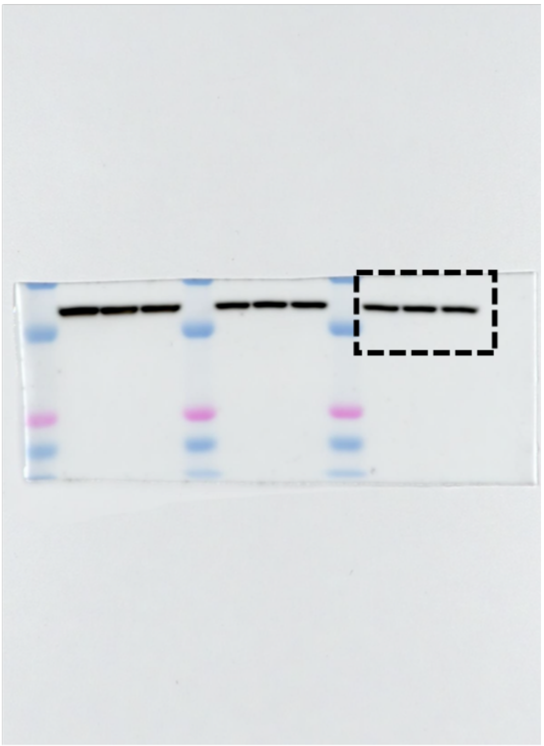

Supplement: S1 Raw image — (PDF) [file pone.0274829.s002.pdf]
